# DEPRESSION AND ROLE OVERLOAD IN FAMILY CAREGIVERS OF PEOPLE LIVING WITH AND WITHOUT DEMENTIA

**DOI:** 10.1093/geroni/igad104.3267

**Published:** 2023-12-21

**Authors:** Claire McDonald, Adrian Bravo

**Affiliations:** William & Mary, Williamsburg, Virginia, United States; William & Mary, Williamsburg, Virginia, United States

## Abstract

The present study examined the associations between caregiver assistance, role overload as a stressor, and depression symptoms among caregivers of adults age 65 and older living with and without probable dementia. Specifically, we hypothesized that more time spent caregiving would be associated with higher depressive symptoms via greater role overload and this indirect association would be stronger for those caregiving for an older adult with probable dementia versus no probable dementia. This study analyzed data from round 11 of the National Health and Aging Trends Study (NHATS) and the supplemental National Study of Caregiving (NSOC), which were collected from 2021 through early 2022. The majority of caregivers (207 probable dementia caregivers, 451 no probable dementia caregivers) identified as white (69%), female (70%), and reported an average age of 63.33. A moderated mediation model was tested using the PROCESS Macro (Hayes, 2013) in SPSS. Within our model, dementia classification was found to moderate the effect of the number of days spent caring on role overload (B = .048, SE = .019, t = 2.54, p = .011). Moreover, the overall moderated mediation model was supported with the index of moderated mediation = 0.0094 (95% CI = .0010, .0192). These results indicate that the more days a caregiver spent helping was associated with more symptoms of depression via higher caregiver overload, especially among those caregivers taking care of an older adult with probable dementia. These findings highlight the importance of making healthcare services available and accessible for caregivers.

